# Optimal Bayesian estimators for latent variable cluster models

**DOI:** 10.1007/s11222-017-9786-y

**Published:** 2017-10-31

**Authors:** Riccardo Rastelli, Nial Friel

**Affiliations:** 1 Institute for Statistics and Mathematics, WU Vienna University of Economics and Business, Vienna, Austria; 2 School of Mathematics and Statistics, University College Dublin, Dublin, Ireland; 3 Insight: Centre for Data Analytics, Dublin, Ireland

**Keywords:** Bayesian clustering, Cluster analysis, Greedy optimisation, Latent variable models, Markov chain Monte Carlo

## Abstract

In cluster analysis interest lies in probabilistically capturing partitions of individuals, items or observations into groups, such that those belonging to the same group share similar attributes or relational profiles. Bayesian posterior samples for the latent allocation variables can be effectively obtained in a wide range of clustering models, including finite mixtures, infinite mixtures, hidden Markov models and block models for networks. However, due to the categorical nature of the clustering variables and the lack of scalable algorithms, summary tools that can interpret such samples are not available. We adopt a Bayesian decision theoretical approach to define an optimality criterion for clusterings and propose a fast and context-independent greedy algorithm to find the best allocations. One important facet of our approach is that the optimal number of groups is automatically selected, thereby solving the clustering and the model-choice problems at the same time. We consider several loss functions to compare partitions and show that our approach can accommodate a wide range of cases. Finally, we illustrate our approach on both artificial and real datasets for three different clustering models: Gaussian mixtures, stochastic block models and latent block models for networks.

## Introduction

Cluster analysis plays a central role in statistics and machine learning, yet it is not immediately clear how one can appropriately summarise the output of partitions from a Bayesian clustering model. This article seeks to address this impasse, proposing an optimality criterion for clusterings derived from decision theory, and a greedy algorithm to estimate the optimal partition and number of groups. Clustering models are often represented as discrete latent variable models: each of the data objects corresponds to the elements of $\mathcal {V}=\left\{ 1,2,\dots ,N\right\}$ and is characterised by a categorical latent variable $z=\left\{ 1,2,\dots ,K\right\}$ denoting its group label. Such variables are often called *clustering variables* or *allocations*. Notable examples of latent variable clustering models include: product partition models (Hartigan 1990; Barry and Hartigan 1992), finite mixtures (McLachlan and Peel 2004), infinite mixtures (Quintana 2006 and references therein), latent block models for networks (Nowicki and Snijders 2001; Govaert 1995), hidden Markov models (MacDonald and Zucchini 1997).

The motivation for this paper ensues from the introduction within the statistical community of the so-called trans-dimensional samplers. One well-known and widely used sampler is the *reversible jump algorithm* of Green (1995), extended to the context of finite mixtures by Richardson and Green (1997) and to hidden Markov models by Robert et al. (2000). Reversible jump Markov chain Monte Carlo allows one to explore a number of models with a single Markov chain that “jumps” between them, thereby estimating both the model parameters and the posterior model probabilities. A more recent trans-dimensional Markov chain Monte Carlo algorithm is the *allocation sampler* introduced by Nobile and Fearnside (2007). This takes advantage of the fact that, in some mixture models, the marginal posterior distribution of the allocation variables can be obtained by analytically integrating out all of the model parameters. This allows one to use a *collapsed* Gibbs sampler and obtain a posterior marginal sample for the clustering variables. One advantage of this method is that the number of groups can be inferred at each step from the clustering variables automatically, hence obtaining posterior probabilities for the different models. The core idea of the allocation sampler has been recently extended to a number of frameworks, including latent class analysis (White et al. 2016), latent block models (Wyse and Friel 2012), stochastic block models (McDaid et al. 2013), latent position models (Ryan et al. 2017) and change point analysis (Benson and Friel 2016). In Bayesian nonparametrics a similar approach has been proposed by Neal (2000), Favaro and Teh (2013) for Dirichlet process mixture models.

Both reversible jump and allocation sampler return a trans-dimensional sample for the allocations. Theoretically, such a sample contains all of the posterior information needed for the clustering of the data; however, interpreting such information is a very challenging task. Since the allocations are categorical variables, usual summary statistics such as the mean, median and quantiles are not well defined. In addition, these Markov chain Monte Carlo algorithms are sensitive to label-switching issues (Stephens 2000); in fact, when using the latent variable representation, all mixture models are non-identifiable up to a permutation of the cluster labels. In addition, the sample itself may be computationally impractical to handle, since even basic operations may require a cost that grows with $N^2$ or the square of the size of the sample.

The problem described really boils down to a very simple research question: we want to summarise the information provided by a sample of partitions into an optimal partition. This issue has been addressed in several previous works, such as Strehl and Ghosh (2003), Gionis et al. (2007), Dahl (2009), Fritsch and Ickstadt (2009), where the authors propose a number of approaches that define a theoretical optimal partition and introduce algorithms to find it. One critique to these contributions is that the proposed methodologies lack a sound theoretical background and they may be seen as ad hoc.

In this work we use a Bayesian decision theoretical framework to define an optimality criterion for partitions, as previously proposed by Binder (1978), Lau and Green (2007), Wade and Ghahramani (2015). From the Bayesian theoretical point of view, our approach defines the best possible solution to the partitioning problem using the information contained in the sample. Also, an important facet of this methodology is that it builds upon recent adaptations of the allocation sampler (Wyse and Friel 2012; McDaid et al. 2013; Ryan et al. 2017; White et al. 2016), making up for one important shortcoming of these samplers: the interpretation of the results.

The essence of the decision theoretical framework lies in the definition of a loss function in the space of partitions, which is often a metric measuring how different two partitions are. Then, the optimal partition is estimated as the one minimising the average loss with respect to the sample given. In the Bayesian perspective, this is equivalent to adopting a Bayes estimator (or *Bayes action*), which is the decision minimising the *expected posterior loss* (EPL).

We propose a greedy algorithm as means to find the optimal partition, focusing on its computational complexity and scalability. The algorithm can deal with a wide family of loss functions and requires only the sample of partitions as input. Hence, our methodology has wide applicability and is the only scalable procedure that can be used to perform Bayesian clustering for a relatively arbitrary loss function. One important advantage of our algorithmic frameworks is that the resulting optimal clustering automatically determines the optimal number of groups.

Previous works (Lau and Green 2007; Wade and Ghahramani 2015) were confined to the case of Bayesian nonparametric models. Here we stress that this approach is automatically extended to a very general clustering context, and hence, we propose applications to several different frameworks.

The R package GreedyEPL accompanying this paper is publicly available on CRAN, and it contains an implementation of the procedure described.

The plan of the paper is summarised as follows: Sect. 2 describes the theoretical foundations of Bayesian clustering; in Sect. 3 we describe the properties of several loss functions to compare partitions, and we characterise the wide breadth to which our method extends; in Sect. 4 we introduce our greedy algorithm and analyse its complexity and features, whereas Sect. 5 shows an interesting procedure that can be used to potentially save an amount of computational time. We propose a simulation study in Sect. 6, where we assess the efficiency of our method, and we compare its performance with that of other available algorithms. We consider several loss functions and assess their ability to recover the true clustering and the correct model. Finally, three applications to real datasets are proposed in Sect. 7: the Old Faithful dataset for Gaussian mixture models, the French political blogosphere for stochastic block models and the congressional voting data for latent block models. Section 8 closes the paper with some final comments.

## Bayesian clustering: the theory

Let $\mathbf Z$ be a $T\times N$ matrix, where, for every $t=1,\dots ,T$ and $i=1,\dots ,N$, $z_{ti}$ is a categorical variable (typically $z_{ti}\in \left\{ 1,2,\dots ,N\right\}$) indicating the cluster label of observation *i* at iteration *t*. The rows of $\mathbf Z$ determine a sample of partitions of the same set $\mathcal {V} = \left\{ 1,2,\dots ,N\right\}$, and we assume that such sample is drawn from the posterior distribution of a clustering model, given the observed data $\mathcal {Y}$. An alternative representation of the sample would be $\left\{ \mathbf z ^{(1)},\dots ,\mathbf z ^{(T)}\right\}$, where $\mathbf z ^{(t)} = \left\{ z_{t1}, \dots , z_{tN}\right\} \in \mathcal {Z}$ corresponds to the *t*-th row of $\mathbf Z$, and $\mathcal {Z}$ is the space of all partitions of $\mathcal {V}$.

Interest lies in conveying the information provided by the posterior sample into a single optimal partition. Bayesian decision theory offers an elegant approach to tackle this task, essentially recasting the clustering problem into one of decision-making.

The first step consists of choosing a loss function $\mathcal {L}: \mathcal {Z}\times \mathcal {Z} \rightarrow \mathbb {R}$. For any two partitions (hereafter also called *decisions*) $\mathbf a$ and $\mathbf z$, the quantity $\mathcal {L}\left( \mathbf a ,\mathbf z \right)$ indicates the loss occurring when the decision $\mathbf a$ is chosen, while $\mathbf z$ is the correct partition. The choice of the loss function adopted is completely arbitrary and supposedly situational; nonetheless, some loss functions have interesting features and tend to work well in many contexts. A loss function is not necessarily a distance in the space of partitions although this is often regarded as a desirable property, since it helps particularly in the interpretation and representation of the results.

An optimal decision (also called *Bayes action*) is one minimising the *expected posterior loss*, defined as:

1 $$\begin{aligned} \varPsi \left( \mathbf a \right) := \mathbb {E}_\mathbf z \left[ \mathcal {L}\left( \mathbf a ,\mathbf z \right) \vert \mathcal {Y}\right] = \sum _\mathbf{z \in \mathcal {Z}} \pi \left( \mathbf z \vert \mathcal {Y}\right) \mathcal {L}\left( \mathbf a ,\mathbf z \right) . \end{aligned}$$

Considering that the posterior sample $\left\{ \mathbf z ^{(1)},\dots ,\mathbf z ^{(T)}\right\} \sim \pi \left( \ \cdot \ \vert \mathcal {Y}\right)$ is available, for every decision $\mathbf a \in \mathcal {Z}$, an unbiased estimator of the associated expected posterior loss results as:

2 $$\begin{aligned} \psi \left( \mathbf a \right) = \frac{1}{T}\sum _{t=1}^T \mathcal {L}\left( \mathbf a ,\mathbf z ^{(t)} \right) \approx \varPsi \left( \mathbf a \right) . \end{aligned}$$

We aim then at finding the decision $\hat{\mathbf{a }}$ minimising the approximate expected posterior loss:

3 $$\begin{aligned} \hat{\mathbf{a }} = \mathop {{{\mathrm{arg\,\min }}}}\limits _{\mathbf{a \in \mathcal {Z}}} \psi \left( \mathbf a \right) . \end{aligned}$$

## Choice of the loss function

### Common loss functions

Given the sample $\mathbf Z$, a naive but fast method to obtain an optimal clustering would be to consider the single partition that obtained the highest posterior value during the sampling, i.e.:

4 $$\begin{aligned} \hat{\mathbf{a }}_{\mathrm{MAP}} = \mathop {{{\mathrm{arg\,\max }}}}\limits _{{t=1,2,\dots ,T}} \pi \left( \mathbf z ^{(t)}\vert \mathcal {Y} \right) . \end{aligned}$$

In a decision theoretical context, this is equivalent to choosing a 0–1 loss defined as:

5 $$\begin{aligned} \mathcal {L}\left( \mathbf a ,\mathbf z \right) = \left\{ \begin{array}{ll} 1&{}\quad \text{ if } {} \mathbf a \not \equiv \mathbf z ,\\ 0&{}\quad \text{ if } {} \mathbf a \equiv \mathbf z ; \end{array}\right. \end{aligned}$$

since the Bayes action minimising (3) would simply be the mode of the sample. The sign “$\equiv$” here means that there exists a label permutation $\sigma$ such that $\sigma \left( a_i \right) = z_i$, $\forall i\in \mathcal {V}$. Reading the definition in (5), the loss is zero iff the partitions are equivalent. In all of the other cases, the loss is 1 regardless of how different the partitions actually are. This peculiar behaviour makes the 0–1 loss rather unappealing as means to compare partitions. Note that all of the clustering algorithms that return a MAP estimate can be interpreted in this context as tools minimising the expected 0–1 loss, although they normally do not require the sample $\mathbf Z$, and are very computationally efficient. Hence, MAP estimates may be criticised since in the Bayesian paradigm the corresponding loss is not particularly sensible.

Another loss function that is commonly used is the quadratic loss, which gives the posterior mean as Bayes action. However, in a clustering context this has little meaning due to the categorical nature of the variables, which makes any sort of averaging of allocations not particularly meaningful.

### Loss functions to compare partitions

A more sensible approach would be to choose a loss function that is specifically designed to compare partitions. In recent years, many measures to compare partitions have been proposed, each with very different properties and characteristics. The works of Meilă (2007), Vinh et al. (2010), Wade and Ghahramani (2015) and references therein offer an excellent overview.

A common approach used to compare partitions (here $\mathbf a$ and $\mathbf z$ denote two arbitrary partitions with $K_\mathbf{a }$ and $K_\mathbf{z }$ groups, respectively) relies on the $K_\mathbf{a } \times K_\mathbf{z }$ contingency matrix (or confusion matrix), whose entries are defined as:

6 $$\begin{aligned} n^\mathbf{a ,\mathbf z }_{gh} = \sum _{i=1}^{N} \mathbb {1}_{\left\{ a_i=g\right\} }\mathbb {1}_{\left\{ z_i=h\right\} } \end{aligned}$$

where *g* varies among the groups of $\mathbf a$, and *h* among those of $\mathbf z$. The entries of such a matrix simply count the number of items that $\mathbf a$ classifies in group *g* and $\mathbf z$ classifies in group *h*, for every *g* and *h*.

Here, we focus on loss functions that depend on $\mathbf a$ and $\mathbf z$ only through the entries of $\mathbf n ^\mathbf{a ,\mathbf z }$. This is a fairly general and reasonable assumption which is in line with the theory developed by Binder (1978); in fact, most metrics can be transformed into functions of the counts (see Vinh et al. 2009 and references therein).

We assume that the loss function has the following representation:

7 $$\begin{aligned}&\mathcal {L}\left( \left\{ n^\mathbf{a ,\mathbf z }_{gh}\right\} _{g,h}, \left\{ n^\mathbf{a }_{g}\right\} _{g}, \left\{ n^\mathbf{z }_{h}\right\} _{h} \right) \nonumber \\&\quad = f_0\left( \sum _{g=1}^{K_\mathbf{a }}\sum _{h=1}^{K_\mathbf{z }} f_1\left( n^\mathbf{a ,\mathbf z }_{gh} \right) , \sum _{g=1}^{K_\mathbf{a }} f_2\left( n^\mathbf{a }_{g} \right) , \sum _{h=1}^{K_\mathbf{z }} f_3\left( n^\mathbf{z }_{h} \right) \right) \nonumber \\ \end{aligned}$$

where $f_0,\ f_1,\ f_2,\ f_3$ are real-valued functions that can be evaluated in constant time and $n^\mathbf{a }_{g}$ and $n^\mathbf{z }_{h}$ indicate the sizes of group *g* and *h*, respectively, i.e.:

8 $$\begin{aligned} n^\mathbf{a }_{g} = \sum _{h=1}^{K_\mathbf{z }} n^\mathbf{a ,\mathbf z }_{gh}, \quad n^\mathbf{z }_{h} = \sum _{g=1}^{K_\mathbf{a }} n^\mathbf{a ,\mathbf z }_{gh}. \end{aligned}$$

for every $g=1,\dots ,K_\mathbf{a }$ and $h=1,\dots ,K_\mathbf{z }$. The assumption determined by (7) is actually not restrictive: most of the commonly used loss functions for partitions satisfy this condition. We note that the arguments of the function $f_0$ include the following quantities as special cases:

- The entropies of $\mathbf a$ and $\mathbf z$, describing the uncertainty associated with $\mathbf a$ and $\mathbf z$, respectively:
  9 $$\begin{aligned} H\left( \mathbf a \right)= & {} -\sum _{g=1}^{K_\mathbf{a }} \frac{n^\mathbf{a }_{g}}{N} \log _2 \frac{n^\mathbf{a }_{g}}{N};\nonumber \\ H\left( \mathbf z \right)= & {} -\sum _{h=1}^{K_\mathbf{z }} \frac{n^\mathbf{z }_{h}}{N} \log _2\frac{n^\mathbf{z }_{h}}{N}. \end{aligned}$$
- The joint entropy of $\mathbf a$ and $\mathbf z$:
  10 $$\begin{aligned} H\left( \mathbf a ,\mathbf z \right) = -\sum _{g=1}^{K_\mathbf{a }}\sum _{h=1}^{K_\mathbf{z }} \frac{n^\mathbf{a ,\mathbf z }_{gh}}{N}\log _2\frac{n^\mathbf{a ,\mathbf z }_{gh}}{N}. \end{aligned}$$
  This describes instead the uncertainty of the random variable with probability density function given by the quantities $n^\mathbf{a ,\mathbf z }_{gh}/N$, for every *g* and *h*.
- The mutual information, which can be evaluated from the entropies and joint entropy:
  11 $$\begin{aligned} I\left( \mathbf a ,\mathbf z \right) = H\left( \mathbf a \right) + H\left( \mathbf z \right) - H\left( \mathbf a ,\mathbf z \right) . \end{aligned}$$
  This quantity is particularly meaningful and has been advocated in a normalised version by Strehl and Ghosh (2003) as a distance measure between partitions.

Note the common convention that $x\log _2 x = 0$ if $x=0$. Evidently these information-based quantities can be obtained as special cases of the functions $f_1$, $f_2$ and $f_3$, making our assumption rather general and broadly satisfied.

Here follows a brief description of some well-known loss functions that are included in our framework. *Binder’s loss (B)* We use a special case of a more general formula first introduced by Binder (1978):

12 $$\begin{aligned} \mathcal {L}_{\mathrm{B}}\left( \mathbf a ,\mathbf z \right) = \frac{1}{2}\sum _{g=1}^{K_\mathbf{a }} \left( n^\mathbf{a }_g\right) ^2 + \frac{1}{2}\sum _{h=1}^{K_\mathbf{z }} \left( n^\mathbf{z }_h\right) ^2 - \sum _{g=1}^{K_\mathbf{a }}\sum _{h=1}^{K_\mathbf{z }} \left( n^\mathbf{a ,\mathbf z }_{gh}\right) ^2. \end{aligned}$$

This loss is equivalent to the Hamming distance (Meilă 2012) and to the Rand index (Rand 1971). Binder’s loss has an interesting property that simplifies greatly the minimisation of (3). One can in fact easily construct a so-called posterior similarity matrix of size $N\times N$, whose entries $b_{ij}$ denote the estimated posterior probability of *i* and *j* being allocated to the same group, for every *i* and *j* in $\mathcal {V}$. Then, the Binder Bayes action satisfies:

13 $$\begin{aligned} \hat{\mathbf{a }}_\mathrm{B} = \mathop {{{\mathrm{arg\,\min }}}}\limits _{\mathbf{a \in \mathcal {Z}}}\sum _{i<j}\left[ \mathbb {1}_{\left\{ a_i = a_j\right\} } - b_{ij}\right] \end{aligned}$$

where $\mathbb {1}_\mathcal {A}$ is equal to 1 if the event $\mathcal {A}$ is true or zero otherwise. This simplifies the minimisation problem since (13) depends on the sample only through the posterior similarity matrix, which can be effectively computed beforehand.

*The variation of information (VI)* This loss is one we particularly focus on in this paper and is defined as:

14 $$\begin{aligned} \mathcal {L}_{\mathrm{VI}}\left( \mathbf a ,\mathbf z \right) = 2H\left( \mathbf a ,\mathbf z \right) - H\left( \mathbf a \right) - H\left( \mathbf z \right) . \end{aligned}$$

The VI loss, first studied in Meilă (2007), has received an increasing amount of attention in the last decade, mainly due to its strong mathematical foundations and practical efficiency. In the paper by Meilă (2007) as well as in subsequent works such as Wade and Ghahramani (2015), the mathematical properties and behaviour of the VI loss have been deeply studied. We mention that this loss is a metric, that it forms a lattice and that it is horizontally and vertically aligned in the space of partitions. In addition, it is invariant to label-switching, i.e. switching labels for either $\mathbf a$ or $\mathbf z$ will not affect the value $\mathcal {L}_{\mathrm{VI}}\left( \mathbf a ,\mathbf z \right)$. More details regarding the theoretical properties of the VI loss can be found in Meilă (2007).

*The normalised variation of information (NVI)* This loss is defined as:

15 $$\begin{aligned} \mathcal {L}_{\mathrm{NVI}}\left( \mathbf a ,\mathbf z \right) = 1 - \frac{I\left( \mathbf a ,\mathbf z \right) }{H\left( \mathbf a ,\mathbf z \right) }. \end{aligned}$$

The normalised version of the VI loss takes values in [0, 1]. This scale-invariance may facilitate the interpretation and the comparisons of partitions under different conditions. Since we adopt an optimisation approach, this feature is not crucial in our framework due to the partitions always referring to the same set of individuals.

*The normalised information distance (NID)* This loss is defined as:

16 $$\begin{aligned} \mathcal {L}_{\mathrm{NID}}\left( \mathbf a ,\mathbf z \right) = 1-\frac{I\left( \mathbf a ,\mathbf z \right) }{\max \left\{ H\left( \mathbf a \right) ,H\left( \mathbf z \right) \right\} }. \end{aligned}$$

The NID loss has been advocated in Vinh et al. (2010) as a general purpose—context independent—loss function with desirable behaviours.

## Minimisation of the expected posterior loss

An exhaustive search within $\mathcal {Z}$ becomes impractical even for very small *N* (the cardinality of $\mathcal {Z}$ is a number with more than 100 digits if $N=100$). Therefore, the minimisation can be seen as a binary programming optimisation problem which is known to be NP-hard, and hence not solvable through exact methods.

Also, the objective function requires the calculation of the sum in (2) at each evaluation. Getting a new posterior sample at each step is not a practical option; hence, the same sample is used for all of the evaluations of (2). Nonetheless, even a single evaluation of the objective function can become computationally burdensome when the size of the sample is large. Therefore, the decision theoretical approach becomes soon impractical as *N* and *T* increase, and finding scalable procedures is crucial. In this section we introduce a new algorithm that, using greedy updates, is able to estimate the Bayes action for the wide family of loss functions satisfying (7), requiring in input only the posterior sample of partitions.

### Greedy algorithm

Heuristic greedy algorithms have been recently rediscovered as a means to maximise the so-called *exact integrated complete likelihood* in various contexts: stochastic block models (Côme and Latouche 2015), latent block models (Wyse et al. 2017), Gaussian finite mixtures (Bertoletti et al. 2015). Similar approaches have also been proposed in Bayesian nonparametrics for Dirichlet prior mixtures (Raykov et al. 2016) although in this case they did not cast the clustering problem into the optimisation of an exact model-based clustering criterion. Among the many papers adopting types of greedy optimisation, we find the approaches of Besag (1986), Strehl and Ghosh (2003), Newman (2004) particularly related to ours.

We propose a greedy algorithm that updates a partition by changing the cluster memberships of single observations using a greedy heuristic, hence decreasing the expected posterior loss of the partition at each step. As input, the algorithm only requires a starting partition, the posterior sample $\mathbf Z$ and a user-specified parameter $K_{\mathrm{up}}$, equal to the maximum number of groups allowed (a reasonable default value would be $K_{\mathrm{up}}=N$). The algorithm cycles over the observations in random order, and, for each of these, it tries all of the possible reallocations, eventually choosing the one giving the best decrease in the objective function. The notation $\mathbf a _{i:r\rightarrow s}$ denotes the partition $\mathbf a$ where the observation *i* has been reallocated from group *r* to *s*. At each move, the number of groups may increase (if the observation is reallocated to an empty group) or decrease (if a group is left empty), although the latter scenario is much more frequent. Due to the low probability of creating new groups, it is generally advisable to start with a partition made of close to $K_{\mathrm{up}}$ groups. The procedure stops when a complete sweep over all observations yields no change in the expected posterior loss. The pseudo-code for the algorithm is shown in Algorithm 1. 
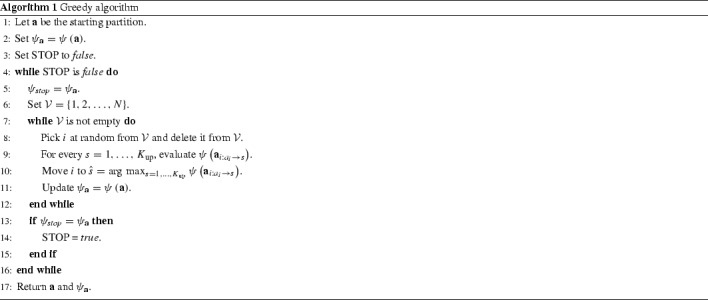


Due to the greedy nature of this procedure, the algorithm is bound to return a local optimum, rather than a global one. Consequently, several restarts with different initial partitions may be required. However, due to the greedy behaviour, convergence is usually reached in very few iterations, in each run. In all of the applications proposed we choose the starting partition completely at random among those with up to $K_{\mathrm{up}}$ groups. Since the algorithm has a tendency to reduce the number of groups, we normally set $K_{\mathrm{up}}$ to a large value (as large as computational resources allow for), which gives the best chances to converge to a global optimum. By contrast, choosing, for example, the MAP as starting partition may result in premature convergence and local optima issues. We note that these indications are in line with those provided in Hastie et al. (2015).

One interesting feature of the greedy algorithm is that the whole space of partitions is explored; hence, the optimal partitions may differ substantially from all of the clusterings in the sample. In fact, many non-optimal solutions may have higher posterior values than the optimal one. In contrast to Côme and Latouche (2015), Wyse et al. (2017), we do not perform any final merge step, as in most cases this did not improve the results.

### Complexity

The basic operation that determines the complexity of the greedy optimisation is the evaluation of the variation in the objective function when a possible reallocation is tested (line 9 in the pseudo-code 1). Assume that the move from $\mathbf a$ to $\mathbf a _{i:r\rightarrow s}$ is being tested, for some groups *r* and *s*. The following quantity needs to be evaluated:

17 $$\begin{aligned} \Delta \psi:= & {} \psi \left( \mathbf a _{i:r\rightarrow s} \right) - \psi \left( \mathbf a \right) \nonumber \\= & {} \frac{1}{T}\sum _{t=1}^{T} \left[ \mathcal {L}\left( \mathbf a _{i:r\rightarrow s},\mathbf z ^{(t)} \right) - \mathcal {L}\left( \mathbf a ,\mathbf z ^{(t)} \right) \right] , \end{aligned}$$

which in turn requires, $\forall t= 1,\dots , T$:

18 $$\begin{aligned} \Delta \mathcal {L}^{(t)} := \mathcal {L}\left( \mathbf a _{i:r\rightarrow s},\mathbf z ^{(t)} \right) - \mathcal {L}\left( \mathbf a ,\mathbf z ^{(t)} \right) . \end{aligned}$$

For a certain *t*, the move only affects two entries of $\mathbf n ^\mathbf{a }$ (i.e. $n^\mathbf{a }_{r}$ and $n^\mathbf{a }_{s}$) and two entries of $\mathbf n ^\mathbf{a ,\mathbf z ^{(t)}}$ (i.e. $n^\mathbf{a ,\mathbf z ^{(t)}}_{rv}$ and $n^\mathbf{a ,\mathbf z ^{(t)}}_{sv}$, where $v = z_{ti}$). This means that the change in the arguments of $f_0$ can be evaluated in a constant time, hence making the cost of evaluating $\Delta \psi \sim \mathcal {O}\left( T \right)$.

Since the algorithm tries all possible moves for each observation, the overall computational cost is $\mathcal {O}\left( TNK_{\mathrm{up}} \right)$.

### Comparisons with other algorithms

Both Lau and Green (2007) and Wade and Ghahramani (2015) proposed original algorithmic frameworks to minimise an expected posterior loss. While Lau and Green (2007) only focused on Binder’s loss, Wade and Ghahramani (2015) also extended the procedure to the VI loss, albeit resorting to an approximation of the objective function. Both methodologies take advantage of the posterior similarity matrix representation, briefly pointed out in (13). Note that this representation is exclusive to the Binder’s loss; hence, these approaches lack the possibility to be generalised to other loss functions, unless approximations are introduced.

The computational cost for an evaluation of the objective function (13) does not depend on *T*, since the posterior information contained in the sample is summarised in the posterior similarity matrix. The calculation of the posterior similarity matrix itself requires $\mathcal {O}\left( TN^2 \right)$ operations, yet this can be performed offline and it is unlikely to impact the overall computing time.

On the other hand, our algorithm does not require a $N^2$ cost at any stage; hence, it should be preferable when the number of observations to classify is very large. We note that, due to the dependence of the complexity on *T*, our algorithm will benefit if the sample is small and thinned with a large lag. A trade-off between the reliability of the posterior sample and computing time should be assessed, in which one should provide a sample that is as small as possible but not so small that the approximation to the posterior distribution is not reliable. As concerns $K_{\mathrm{up}}$, in order to minimise the chances to stop at a local optimum, this should be set to a value as large as the computational power allows for: $K_{\mathrm{up}}=N$ would be ideal, but this may only be feasible when *N* is very small.

More generally, the computational cost of the algorithm may be compared to the complexity of the sampler used to get the posterior sample. In fact, one key advantage of the collapsed Gibbs samplers proposed in Nobile and Fearnside (2007), McDaid et al. (2013), Wyse and Friel (2012) is their computational efficiency. The posterior sample returned by these samplers is necessary to perform the minimisation of the expected posterior loss. Hence, an ideal complexity for the optimisation problem should be not higher than that required by the sampler in the first place. Unfortunately, when analysing these samplers, new quantities (the number of dimensions for Gaussian mixtures, or the number of edges in block models) come into play, making a strict comparison of the complexity not possible. However, in our applications we noticed that the computational bottleneck was always set by the samplers, and not by the greedy algorithm.

## Classes of equivalences in the posterior sample

Since the sample space $\mathcal {Z}$ is discrete, the posterior sample $\mathbf Z$ may contain repetitions, due to the sampler returning to the same partition during the sampling procedure. This suggests that, regardless of the partition $\mathbf a$, a number of the calculations required to obtain $\mathcal {L}\left( \mathbf a ,\mathbf z \right)$ are redundant. In fact, given a partition $\mathbf z$, the following holds:

19 $$\begin{aligned}&\mathcal {L}\left( \mathbf a ,\mathbf z ^{(t)} \right) = \mathcal {L}\left( \mathbf a ,\mathbf z \right) ;\end{aligned}$$

20 $$\begin{aligned}&\mathcal {L}\left( \mathbf a _{i\rightarrow g},\mathbf z ^{(t)} \right) = \mathcal {L}\left( \mathbf a _{i\rightarrow g},\mathbf z \right) . \end{aligned}$$

for all $i=1,\dots ,N$ and $g=1,\dots ,K_{\mathrm{up}}$ and $\forall t:\ \mathbf z ^{(t)} \equiv \mathbf z$.

It follows that the posterior sample can be summarised into the sample of its unique rows $\tilde{\mathbf{Z }} = \left\{ \tilde{\mathbf{z }}^{(1)},\dots ,\tilde{\mathbf{z }}^{(\tilde{T})}\right\}$ and a vector of counts $\varvec{\omega } = \left\{ \omega ^{(1)},\dots ,\omega ^{(\tilde{T})}\right\}$ describing how many times the corresponding partition appears in the original sample $\mathbf Z$. Therefore, the approximate expected posterior loss can be equivalently written as:

21 $$\begin{aligned} \psi \left( \mathbf a \right) = \frac{1}{\tilde{T}}\sum _{t=1}^{\tilde{T}} \omega ^{(t)}\mathcal {L}\left( \mathbf a ,\tilde{\mathbf{z }}^{(t)} \right) . \end{aligned}$$

A similar reasoning can be used to make the calculation of $\psi \left( \mathbf a _{i\rightarrow g} \right)$ more efficient.

The main difficulty in applying the technique just described lies in identifying the new representation efficiently. One problem consists in the implementation of the operator “$\equiv$” since partitions should be compared up to a permutation of the labels. To solve this, we use a procedure described in Strehl and Ghosh (2003) that defines a unique labelling for all partitions: the first item is assigned to cluster 1, and then iteratively the next item is assigned either to an existing cluster or to the next empty cluster. Using this relabelling, any two equivalent partitions will be transformed into the same sequence of digits in a computational time $\mathcal {O}\left( TN \right)$.

Furthermore, the same vector can be seen as a number in base—$K_{\mathrm{up}}$ representation which uniquely identifies the corresponding partition and the equivalence class imposed by “$\equiv$”. Hence, a sorting algorithm can be used to reorder the sample according to such identifiers, for a computational cost of $\mathcal {O}\left( NT\log T \right)$, where *N* is the cost of a single comparison of partitions. Once the partitions are sorted, the unique set and the corresponding weights can be obtained in $\mathcal {O}\left( TN \right)$.

The advantage provided by this representation heavily depends on the dataset and on the corresponding marginal posterior distribution: less repetitions will appear if the posterior is flat and the partitioning very uncertain. On the other hand, the computational savings may be substantial in cases where only few partitions have a high posterior value.

Note that the sorting procedure creates a new computational bottleneck in the case where $\log T > K_{\mathrm{up}}$. However, we found this is not relevant in practical terms and negligible when compared to the computational time demanded by the actual optimisation.

In a machine learning context, the weighted sample $\tilde{\mathbf{Z }}$ may be interpreted as a cluster ensemble problem, whereby each partition corresponds to the output of a clustering algorithm and the counts are weights describing the relative (possibly subjective) importance of the solution. Our methodology may be applied in this scenario without further modifications, providing a sound background to the decision-making process.

## Simulations

In this section we propose two applications to simulated bivariate Gaussian mixtures datasets. The design of these experiments resembles that of Wade and Ghahramani (2015).

In a finite mixture model, each data observation $\mathbf y _i \in \mathbb {R}^2$ is generated from a mixture of bivariate Gaussians:

22 $$\begin{aligned} p\left( \mathcal {Y}\vert \varvec{\lambda }, \varvec{\mu },\varvec{\Sigma } \right) = \prod _{i=1}^{N}\sum _{g=1}^{K}\lambda _g\mathcal {MVN}_2\left( \mathbf y _i;\ \varvec{\mu }_g, \varvec{\Sigma }_g \right) ; \end{aligned}$$

where $\lambda _1,\dots , \lambda _g$ are the mixture weights and $\mathcal {MVN}_2\left( \ \cdot \ ; \varvec{\mu }, \varvec{\Sigma } \right)$ denotes the bivariate Gaussian distribution with mean $\varvec{\mu }$ and covariance matrix $\varvec{\Sigma }$. Alternatively, using the latent variable representation, an allocation variable $z_i$ is associated with each observation, denoting which Gaussian component has generated the corresponding $\mathbf y _i$, resulting as follows:

23 $$\begin{aligned} p\left( \mathcal {Y}\vert \mathbf z ,\varvec{\mu }, \varvec{\Sigma } \right) = \prod _{g=1}^{K} \prod _{i: z_i=g} \mathcal {MVN}_2\left( \mathbf y _i;\ \varvec{\mu }_g,\ \varvec{\Sigma }_g \right) . \end{aligned}$$

In this section, a Dirichlet process is used as a prior on the model parameters, following the approach of Ferguson (1973). This leads to an *infinite* mixture model which generalises that of (22), since it potentially has an infinite number of mixture components *K*. This generative process assumes that observations are sequentially allocated either to an existing group (with probability $\frac{N_g}{\alpha + N - 1}$, proportional to the size of the corresponding group *g*) or to a new group (with probability $\frac{\alpha }{\alpha + N - 1}$). Each time a new group is created, its specific parameters are generated from:

24 $$\begin{aligned} \begin{aligned}&\varvec{\Sigma }_g \sim \hbox {Inverse Wishart}\left( \nu , u\nu \mathbf I \right) ;\\&\left. \varvec{\mu }_g\vert \varvec{\Sigma }_g\right. \sim \mathcal {MVN}_2\left( \mathbf 0 ,\frac{1}{a}\varvec{\Sigma }_g \right) . \end{aligned} \end{aligned}$$

where $\mathbf I$ denotes the identity matrix of dimension 2. The prior on $\alpha$ is:

25 $$\begin{aligned} p(\alpha ) = \left[ 1-\frac{\left( \alpha -\alpha _{\mathrm{min}} \right) }{\left( \alpha _{\mathrm{max}}-\alpha _{\mathrm{min}}\right) }\right] ^h. \end{aligned}$$

We use the function rDPGibbs from the R package bayesm to obtain posterior samples for the allocation variables. Uniform priors are assumed on the parameters *a*, $\nu$ and *u*, and all of the hyperparameters of the model are set to the default values of rDPGibbs. Once a sample of partitions is obtained, we consider the following algorithms to find the best clustering for the data observations:

- **L&G**: this denotes the algorithm of Lau and Green (2007), as implemented in the function minbinder of mcclust. This algorithm minimises the expected posterior Binder’s loss.
- **W&G**: this denotes the algorithm of Wade and Ghahramani (2015), as implemented in the function minVI of mcclust.ext. This algorithm minimises the expected posterior VI loss, but it relies on a Jensen’s inequality to take advantage of the posterior similarity matrix formulation.
- **MEDV**: this denotes the algorithm of Medvedovic et al. (2004) (implemented in mcclust) where hierarchical clustering is used on the dissimilarity matrix with entries $1-b_{ij}$.
- **PEAR**: this denotes the algorithm of Fritsch and Ickstadt (2009) implemented in mcclust. In this case the algorithm minimises the expected posterior adjusted Rand index.
- **GreedyVI**, **GreedyB**, **GreedyNVI**, **GreedyNID**: these correspond to the optimal partitions obtained using our method, and they are all implemented in the package GreedyEPL accompanying this paper. The corresponding loss functions are the variation of information, Binder’s, normalised variation of information and normalised information distance, respectively. Note that in all of the **Greedy** methods the algorithms are started with a random partition of $K_{\mathrm{up}} = 20$ groups, unless stated otherwise.

### Example 1

The data $\mathcal {Y}$ used in this sample consist of $N = 400$ points drawn from a mixture of $K = 4$ Gaussians where:

26 $$\begin{aligned} \begin{aligned} \lambda _1&= \lambda _2 = \lambda _3 = \lambda _4 = 0.25; \\ \varvec{\mu }_1&= \left( \begin{matrix} 1 \\ 1 \\ \end{matrix}\right) ;\quad \varvec{\mu }_2 = \left( \begin{matrix} -1 \\ 1 \\ \end{matrix}\right) ;\quad \varvec{\mu }_3 = \left( \begin{matrix} -1 \\ -1 \\ \end{matrix}\right) ;\\ \varvec{\mu }_4&= \left( \begin{matrix} 1 \\ -1 \\ \end{matrix}\right) ;\\ \varvec{\Sigma }_1&= \left( \begin{matrix} 1 &{} 0\\ 0 &{} 1 \\ \end{matrix}\right) ;\quad \varvec{\Sigma }_2 = \left( \begin{matrix} 0.5 &{} 0\\ 0 &{} 0.5 \\ \end{matrix}\right) ;\quad \varvec{\Sigma }_3 = \left( \begin{matrix} 1 &{} 0\\ 0 &{} 1 \\ \end{matrix}\right) ;\\ \varvec{\Sigma }_4&= \left( \begin{matrix} 1.5 &{} 0\\ 0 &{} 1.5 \\ \end{matrix}\right) . \end{aligned} \end{aligned}$$

**Fig. 1:**
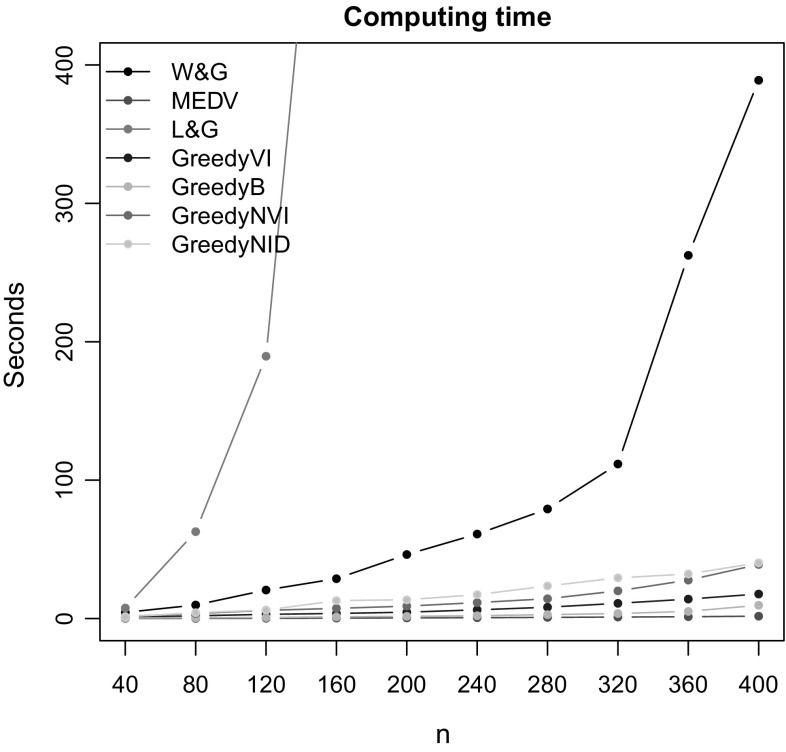
Example 1. Computing times for the clustering methods considered

We aim at studying the scalability of all of the algorithms as *N* increases. First, a posterior sample of 5, 000 partitions is obtained for the full dataset ($N=400$). Then, for *n* varying in $\left( 40,80,120,160,200,240,280,320,360,400 \right)$ each algorithm is run on the subset obtained by selecting only the first *n* observations (and their corresponding allocations). The number of seconds needed for one single run of each of the algorithms is shown in Fig. 1. The method of Lau and Green (2007) scales very poorly with *n* and hence is run only for $n \le 200$. In terms of scalability, the method of Fritsch and Ickstadt (2009) seems to be no different from Medvedovic et al. (2004), and hence, it is not shown in the plot. From the results it appears that the methods based on hierarchical clustering are the fastest, followed by our procedure. The method of Wade and Ghahramani (2015) does not scale particularly well, but it could be improved with a C implementation.

**Fig. 2:**
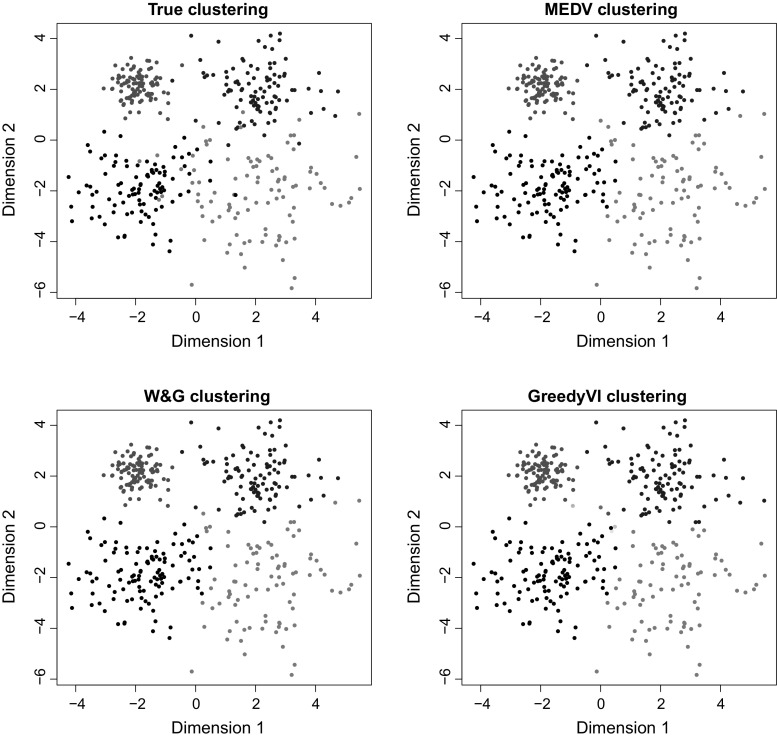
Example 1. True allocations (top-left) and optimal clusterings obtained through **MEDV** (top-right), **W&G** (bottom-left) and **GreedyVI** (bottom-right). It seems that the VI criterion tends to create new groups whenever the allocation of an observation is uncertain

Some of the optimal partitions obtained on the full dataset are shown in Fig. 2. As a measure of quality of the clustering, we consider the number of groups in the optimal partitions (shown in Table 1) and we also compare the optimal partitions to the true one using the variation of information loss, as shown in Table 2. Most algorithms tend to overestimate the number of groups except those of Wade and Ghahramani (2015) and Medvedovic et al. (2004). However, as shown in Table 2, these solutions with 4 groups are not optimal in the VI sense. In fact, the only method minimising the exact expected posterior VI loss is **GreedyVI**, which is also the method achieving the smallest expected posterior VI values. These results suggest that the approximation of Wade and Ghahramani (2015) induces an over-penalisation on the number of groups, whereas most commonly used loss functions (VI included, to some extent) tend to largely overestimate the number of groups.

**Table 1:** Example 1: number of groups for the optimal partitions of each of the methods, for each of the values of *n* considered

	40	80	120	160	200	240	280	320	360	400
W&G	4	4	4	4	4	4	4	4	4	4
MEDV	4	4	4	4	4	4	4	4	4	4
PEAR	5	5	7	7	7	9	12	13	16	17
L&G	8	9	10	11	12					
GreedyVI	6	6	6	5	5	5	7	7	10	10
GreedyB	8	9	10	11	12	15	18	20	24	27
GreedyNVI	9	8	8	7	6	7	10	10	13	16
GreedyNID	7	9	10	12	13	14	16	18	14	21

**Table 2:** Example 1: VI expected posterior loss for each of the optimal partitions with respect to the posterior sample used

	40	80	120	160	200	240	280	320	360	400
W&G	0.860	0.921	0.948	0.994	0.963	0.958	0.978	0.964	1.029	1.054
MEDV	0.893	0.979	1.017	1.025	0.985	0.960	1.024	0.974	1.051	1.055
PEAR	0.840	0.917	0.998	1.014	0.994	0.980	0.996	0.990	1.041	1.064
L&G	0.858	0.986	1.014	1.053	1.036					
GreedyVI	*0.825*	*0.908*	*0.942*	*0.963*	*0.940*	*0.943*	*0.968*	*0.955*	*1.008*	*1.029*
GreedyB	0.858	0.986	1.014	1.053	1.036	1.048	1.067	1.063	1.122	1.161
GreedyNVI	0.864	1.040	0.958	0.971	0.941	0.946	0.974	0.962	1.016	1.162
GreedyNID	0.854	0.954	0.992	1.032	1.009	1.007	1.021	1.020	1.137	1.080

The size of the dataset *n* varies on the columns. The **GreedyVI** method is the only one minimising the non-approximated expected VI loss, and it achieves the smallest values in each dataset (italics)

**Fig. 3:**
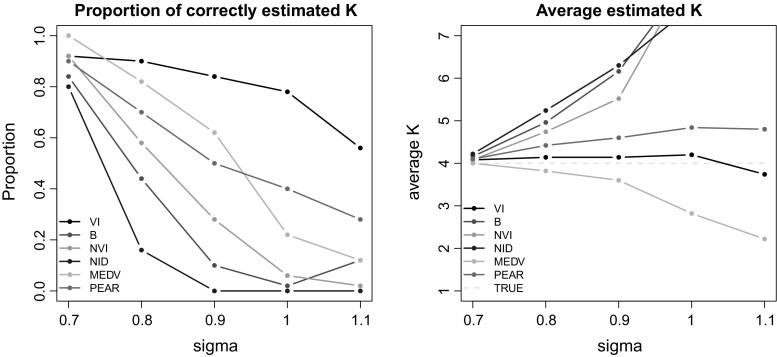
Example 2. On the left panel, the proportion of datasets where the estimated *K* is correct is shown. The average value of *K* is instead shown on the right panel, to assess whether the number of groups was on average underestimated or overestimated

### Example 2

The aim of this study is to assess the performances of the different loss functions considered in Sect. 3.2. The methods **L&G** and **W&G** are not considered since they optimise the same objective functions (or approximation thereof) of our methods **GreedyB** and **GreedyVI** (respectively).

Here we consider the same generative model as in the previous example, but with a different covariance structure:

27 $$\begin{aligned} \varvec{\Sigma }_g = \left( \begin{matrix} \sigma &{} 0 \\ 0 &{} \sigma \\ \end{matrix}\right) \forall g = 1,2,3,4. \end{aligned}$$

The parameter $\sigma$ varies in the set (0.7, 0.8, 0.9, 1, 1.1), and it makes the clustering task more challenging as it increases. For each of these scenarios, 50 datasets of $N = 100$ observations are generated, and a collection of partitions is obtained using the rDPGibbs function of bayesm. The sampler discards the first 25, 000 partitions and then stores one partition every tenth until a sample of length 5, 000 is obtained. For each of these datasets, the algorithms **GreedyVI**, **GreedyB**, **GreedyNVI**, **GreedyNID**, **MEDV** and **PEAR** are run and the corresponding optimal partitions are compared. As concerns model-choice, the results are shown in Fig. 3. Overall, the VI loss achieves the best performance, whereas the methods based on hierarchical clustering over-perform the methods based on the other loss functions. The only method inclined towards an underestimation of the number of groups is that of Medvedovic et al. (2004), confirming the results of Sect. 6.1. All of the other methods tend to overestimate *K*, to some extent. We remark that Binder’s loss, similarly to the normalised variation of information and the normalised information distance, dramatically overestimates the number of groups as the dataset becomes more challenging. These findings are in agreement with those of Wade and Ghahramani (2015).

**Fig. 4:**
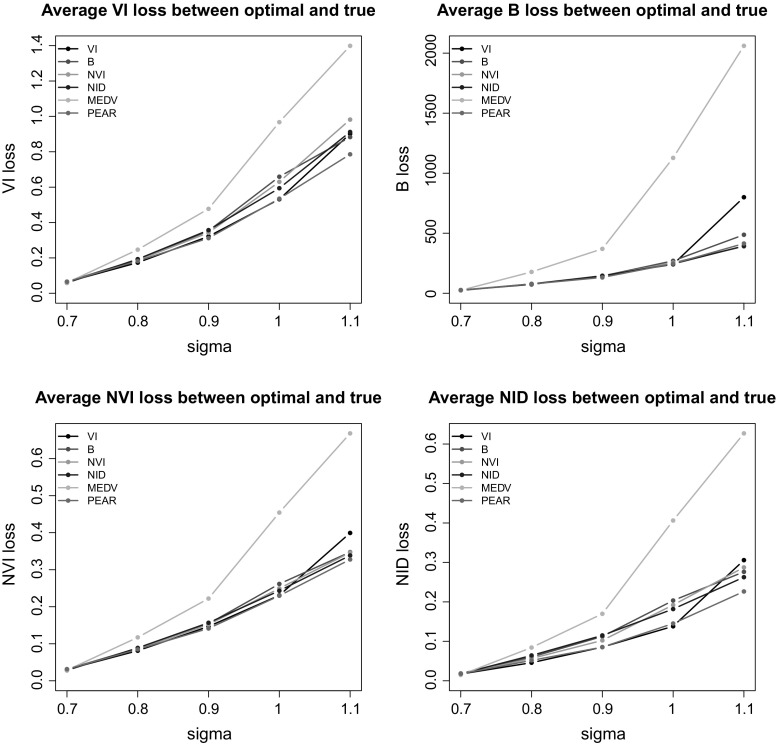
Example 2. All of the optimal partitions are compared to the true one using the following loss functions: variation of information (top-left), Binder’s (top-right), normalised variation of information (bottom-left), normalised information distance (bottom-right)

We also show several comparisons of the optimal clusterings with respect to the true allocations in Fig. 4, using the same loss functions as measures of disagreement. According to these comparison measures, it is clear that the **MEDV** method performs quite poorly, whereas **PEAR** achieves good results in all cases. Regarding the other methods, it seems difficult to rank them. In most cases each method achieves the best scores when it is assessed using the corresponding loss function. The reason why this does not happen every time is simply because the true partition may not necessarily correspond to the best clustering of the data and should not be expected to correspond to the minimum value of any of the objective functions considered. Combining Figs. 3 and 4, it is worth noting that the VI loss has a tendency to overestimate the number of groups when the dataset is not challenging, but it can fail to see any clustering structure if the noise in the data is larger. This is signalled by the fact that its performance declines as the clustering task becomes more difficult.

## Real data examples

In this section, we provide three applications of our methodology to different clustering contexts and compare the results obtained with previous analyses. In light of the results of the previous section, and to simplify the illustration, we only show the results only for the VI loss.

**Fig. 5:**
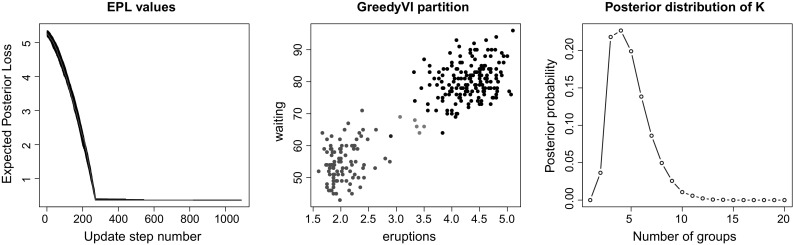
Old Faithful dataset. On the left panel the expected posterior loss values obtained during the optimisation are shown. The best value is reached in all of the 100 runs of the algorithm, suggesting an excellent performance. The red line shows the EPL value evolution for the first run returning the best partition. On the central panel, the VI-optimal partition is shown. The right panel shows instead the posterior probabilities for the number of groups. This distribution has a peak at $K=4$, which corresponds to the number of groups in the **GreedyVI** partition. (Color figure online)

### Old Faithful geyser dataset

#### The data

The dataset considered contains the waiting times between eruptions and the duration of the eruption for the Old Faithful geyser in Yellowstone National Park, Wyoming, USA. Interest lies in predicting the time until the next eruption. The number of observations is $N = 272$. The dataset has been analysed in a number of works, including Azzalini and Bowman (1990), and has been reproposed in many papers dealing with mixture models. The data, shown in Fig. 5, clearly exhibit two regimes: a short eruption will make the wait until the next event shorter, whereas a long eruption will be followed by a longer wait.

#### Results

We obtained a sample for the allocations using the function rDPGibbs from the R package bayesm. One million observations were first discarded as burn-in, and then, one observation every hundredth was retained until a sample size of 10, 000 was obtained. Then, 100 runs of the greedy algorithm were performed, using random starting partitions with $K_{\mathrm{up}} = 20$ groups. The evolution of the expected posterior losses for each of the runs is shown on the left panel of Fig. 5. In this case, the performance of the greedy optimiser is excellent, in that in each single run the algorithm converges to the same optimal partition. The central panel of Fig. 5 shows the best clustering obtained with **GreedyVI**. When compared to the 2 regimes interpretation, this partition exhibits two additional small groups made of the observations with uncertain allocations. We note that the VI-optimal number of groups corresponds to the modal value of *K* in the posterior sample. The computational time needed to obtain the sample was about 30 minutes, whereas an average of 20 s were required for each run of the greedy algorithm.

### Stochastic block models: French political blogosphere

#### The data

The data first appeared in Zanghi et al. (2008) and consist of a undirected graph where nodes represent political blogs’ websites and edges represent hyperlinks between them. As in Latouche et al. (2011) we focus only on a subset of the original dataset, available in the R package mixer. The data consist of a single-day snapshot of political blogs automatically extracted on 14 October 2006 and manually classified by the “Observatoire Présidentielle project”. The graph is composed of 196 nodes and 1432 edges, and the main political parties are the UMP (French “republican”), UDF (“moderate” party), liberal party (supporters of economic liberalism) and PS (French “democrat”), although 11 different parties appear in total. The observed data are modelled by the adjacency matrix $\mathcal {Y}$ whose entries are defined as follows:

28 $$\begin{aligned} y_{ij} = {\left\{ \begin{array}{ll} 1 &{} \text{ if } \text{ an } \text{ undirected } \text{ edge } \text{ between } \text{ blogs } i\hbox { and }j\hbox { appear; }\\ 0 &{} \text{ otherwise; } \end{array}\right. }\nonumber \\ \end{aligned}$$

for every $$1\le i < j \le N$$.

**Table 3:** French blogs: confusion matrix for the variational partition and the political affiliations

	1	2	3	4	5	6	7	8	9	10	11	12
Cap21	2	0	0	0	0	0	0	0	0	0	0	0
CA	0	0	8	0	0	0	0	0	0	0	0	3
FN-MNR-MPF	4	0	0	0	0	0	0	0	0	0	0	0
Les Verts	5	0	2	0	0	0	0	0	0	0	0	0
PCF-LCR	5	0	1	0	0	0	0	0	0	0	0	0
PCF-LCR	1	0	0	0	0	0	0	0	0	0	0	0
PS	5	0	9	0	0	0	19	18	2	4	0	0
PRG	9	0	1	0	0	0	0	1	0	0	0	0
UDF	0	1	1	0	24	6	0	0	0	0	0	0
UMP	1	24	2	11	2	0	0	0	0	0	0	0
Liberaux	1	0	0	0	0	0	0	0	0	0	24	0

**Fig. 6:**
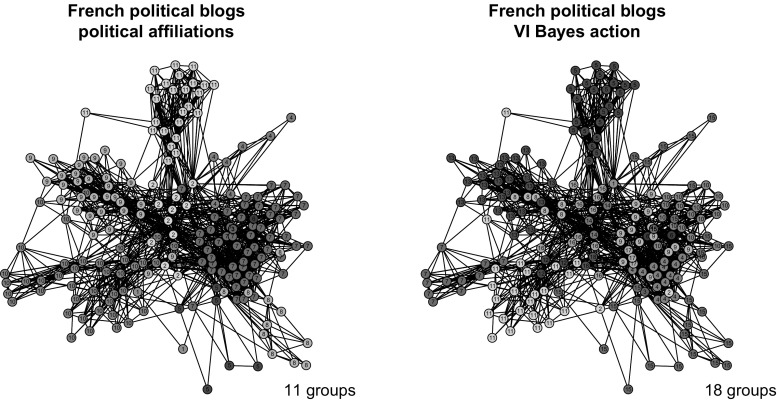
French blogs. Representation of the French political blogs network, with colours and node labels denoting cluster memberships

#### Stochastic block models

Stochastic block models (Nowicki and Snijders 2001) are mixture models for networks, whereby the clustering problem is formulated on the nodes of the network and the connection profile of each node is selected by its cluster membership. For every *i*, the allocation variable $z_i$ denotes the group to which node *i* belongs, and a multinomial-Dirichlet prior structure is assumed on these variables. The number of underlying groups *K* is unknown and hence to be inferred. Conditionally on the allocations, the likelihood for the graph $\mathcal {Y} = \left\{ y_{ij}:\ 1\le i < j \le N\right\}$ factorises as:

29 $$\begin{aligned} P\left( \mathcal {Y}\vert \mathbf z , \Pi \right) = \prod _{g=1}^{K}\prod _{h=1}^{K}\prod _{\left\{ i: z_i = g\right\} }\prod _{\left\{ \begin{array}{c} j: z_j = h \\ j\ne i \end{array}\right\} } \pi _{gh}^{y_{ij}}\left( 1-\pi _{gh} \right) ^{1-y_{ij}}. \end{aligned}$$

Here, $\Pi$ is a symmetric $K\times K$ matrix of connection probabilities, where the generic element $\pi _{gh}$ indicates the probability that an edge occurs between a node in group *g* and a node in group *h*, for any *g* and *h* in $\left\{ 1,\dots ,K\right\}$. Furthermore, each $\pi _{gh}$ is assumed to be a realisation of an independent Beta random variable. The hyperparameters for the Beta and Dirichlet distributions are all set to 0.5.

Since conjugate priors are used, all of the model parameters can be integrated out analytically. It follows that the quantity $p\left( \mathbf z \vert \mathcal {Y} \right)$ is available analytically and can be targeted by a so-called collapsed Gibbs sampler. This is exactly the idea developed by McDaid et al. (2013).

#### Results

First, we performed block modelling using the variational algorithm implemented in the package mixer and obtained a partitioning to be used as reference. The optimal variational solution has 12 groups, which roughly correspond to the political affiliations, as shown in Table 3.

Then, we used our methodology to estimate the VI-optimal partition. The sample for the allocation variables was obtained through the collapsed SBM algorithm of McDaid et al. (2013), discarding the first 1 million updates and keeping 1 observation every 100th thereafter. A sample size of 10, 000 was then used to perform the greedy optimisation, using random starting partitions. The computational time needed to get the sample was about 5 hours, whereas an average of 50 s were required for each run of the greedy algorithm, with $K_{\mathrm{up}}$ fixed to 50. The evolution of the expected posterior losses for each of the runs are shown on the left panel of Fig. 8. We note that the optimal EPL value was actually reached in $10\%$ of the runs.

**Fig. 7:**
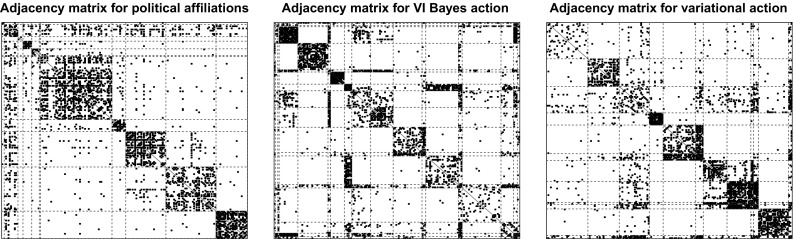
French blogs. Reordered adjacency matrices for three different partitioning of the French political blogs dataset: available political affiliations (left panel), VI-optimal allocations (central panel) and variational optimal allocations (right panel)

**Fig. 8:**
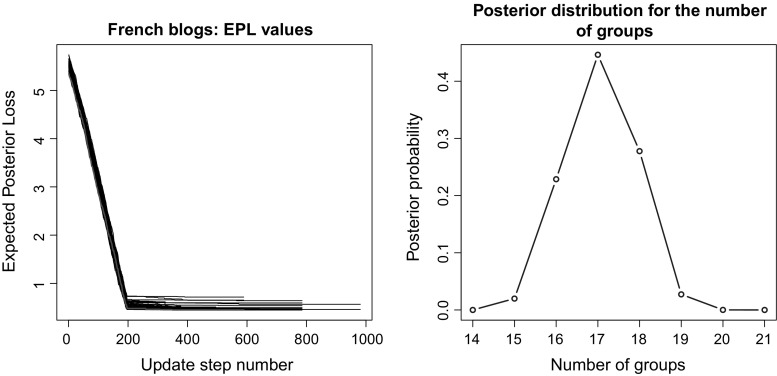
French blogs. On the left panel, the expected posterior loss values obtained during the greedy optimisation are shown. The best value is reached in 10 out of the 100 runs of the algorithm. The red line shows the EPL value evolution for the first run returning the best partition. On the right panel, the posterior distribution for the number of groups in the French political blogosphere dataset is shown. The MAP value is $K=17$ which contrasts with the optimal value obtained through the greedy algorithm. (Color figure online)

The VI-optimal partition exhibits 18 groups and is represented in the right panel of Fig. 6. Figure 7 shows instead the reordered adjacency matrices for the three different partitions. The posterior distribution for the number of groups is shown in Fig. 8. We note that the optimal number of groups contrasts with the modal value of the posterior distribution.

**Table 4:** French blogs: confusion matrix for the VI-optimal partition and the political affiliations

	1	2	3	4	5	6	7	8	9	10	11	12	13	14	15	16	17	18
Cap21	0	0	0	0	0	0	0	0	0	0	0	0	0	0	2	0	0	0
CA	1	0	0	0	0	0	0	0	0	0	0	0	0	3	0	6	0	1
FN-MNR-MPF	0	0	0	0	0	0	0	0	0	0	0	0	0	0	4	0	0	0
Les Verts	0	0	0	0	0	0	0	0	2	0	0	0	0	0	5	0	0	0
PCF-LCR	0	1	0	0	0	0	0	0	0	0	0	0	0	0	5	0	0	0
PCF-LCR	0	0	0	0	0	0	0	0	0	0	0	0	0	0	1	0	0	0
PS	0	0	0	15	0	2	0	0	13	18	0	0	0	0	5	1	3	0
PRG	0	1	1	0	0	0	0	0	0	0	0	0	0	0	9	0	0	0
UDF	0	0	0	0	0	0	0	6	0	0	1	0	24	0	0	0	0	1
UMP	0	0	0	0	0	0	11	0	0	0	21	3	2	0	0	3	0	0
Liberaux	0	0	0	0	24	0	0	0	0	0	1	0	0	0	0	0	0	0

**Fig. 9:**
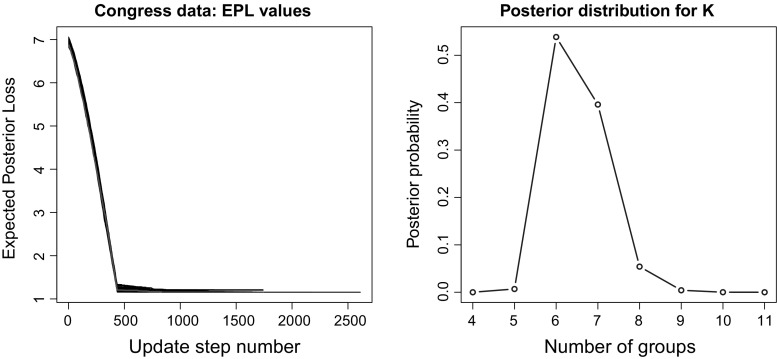
Congressional voting data. On the left panel the expected posterior loss values obtained during the greedy optimisation are shown. The best value is reached in 65 out of the 100 runs of the algorithm. The red line shows the EPL value evolution for the first run returning the best partition. On the right panel, the posterior distribution for the number of groups of congress members is shown. We note that the VI-optimal value $K=6$ corresponds to the modal value. (Color figure online)

It appears that the VI-optimal clustering is a finer partition that splits up some of the larger groups into subgroups. Nonetheless, from Fig. 7 it is clear that this entails a better discrimination of the profiles of blogs. A confusion matrix matching the solution to the political affiliations is shown in Table 4. The liberals are well discriminated in both the variational and VI-optimal partitions. The two partitions also agree on the blogs affiliated to the UDF party: 24 of them are well discriminated and isolated from the rest, a subset of 6 blogs are classified into their own group, 1 blog is associated with the UMP party, and 1 is not well recognised. The main differences between the two partitions arise with respect to the other two relevant parties: UMP and PS. In these two cases it appears that the relational profiles of the blogs are not particularly determined by the political affiliation, since both partitions recognise a number of subgroups within each party, signalling heterogeneity. UMP is decomposed in 5 subgroups in both partitions, while PS is decomposed in 6 and 7 subgroups for the variational and VI partition, respectively.

### Latent block model: congressional voting data

We propose an application of our methodology to the UCI Congressional voting data, previously analysed in Wyse and Friel (2012), Wyse et al. (2017).

#### The data

The data record whether 435 members of the $98^{th}$ congress voted “yay” or “nay” on 16 key issues. Abstained and absent were treated as “nays”. Also, information on the political affiliation of each member is available: 267 individuals are “democrats” and 168 “republicans”. Following Wyse and Friel (2012), Wyse et al. (2017), the data are rearranged into a bipartite network, whereby two types of nodes are defined (one corresponding to congress members and one to issues) and only undirected edges between nodes of different types are allowed. Similarly to stochastic block models, an adjacency matrix $\mathcal {Y}$ is used to summarise the data, with edges corresponding to “yays” ($y_{ij}=1$) and non-edges corresponding to “nays” ($y_{ij}=0$). Note that in this case the matrix $\mathcal {Y}$ has size $435\times 16$, whereby rows correspond to congressmen and columns to issues.

#### Bipartite latent block model

A latent block model (see, for instance, Wyse et al. 2017) is used to model the bipartite graph. A clustering problem is formulated on both the rows and columns of the adjacency matrix: two partitions $\mathbf r$ and $\mathbf c$ determine the clustering of congress members and issues, respectively. The number of groups of $\mathbf r$ and $\mathbf c$ are denoted by $K_r$ and $K_c$, respectively, and are unknown. These two partitions independently follow the same multinomial-Dirichlet structure as described in previous applications.

As concerns the likelihood of the model, a $K_r\times K_c$ matrix $\Pi$ is introduced, so that its generic element $\pi _{gh}\in [0,1]$ corresponds to the probability of the occurrence of an edge from a node in group *g* to a node in group *h*. Hence, conditionally on the allocations, the likelihood can be factorised into independent blocks:

30 $$\begin{aligned} P\left( \mathcal {Y}\vert \mathbf r , \mathbf c , \Pi \right) = \prod _{g=1}^{K_r}\prod _{h=1}^{K_c}\prod _{\left\{ i: r_i = g\right\} }\prod _{\left\{ {j: c_j = h}\right\} } \pi _{gh}^{y_{ij}}\left( 1-\pi _{gh} \right) ^{1-y_{ij}}. \end{aligned}$$

**Fig. 10:**
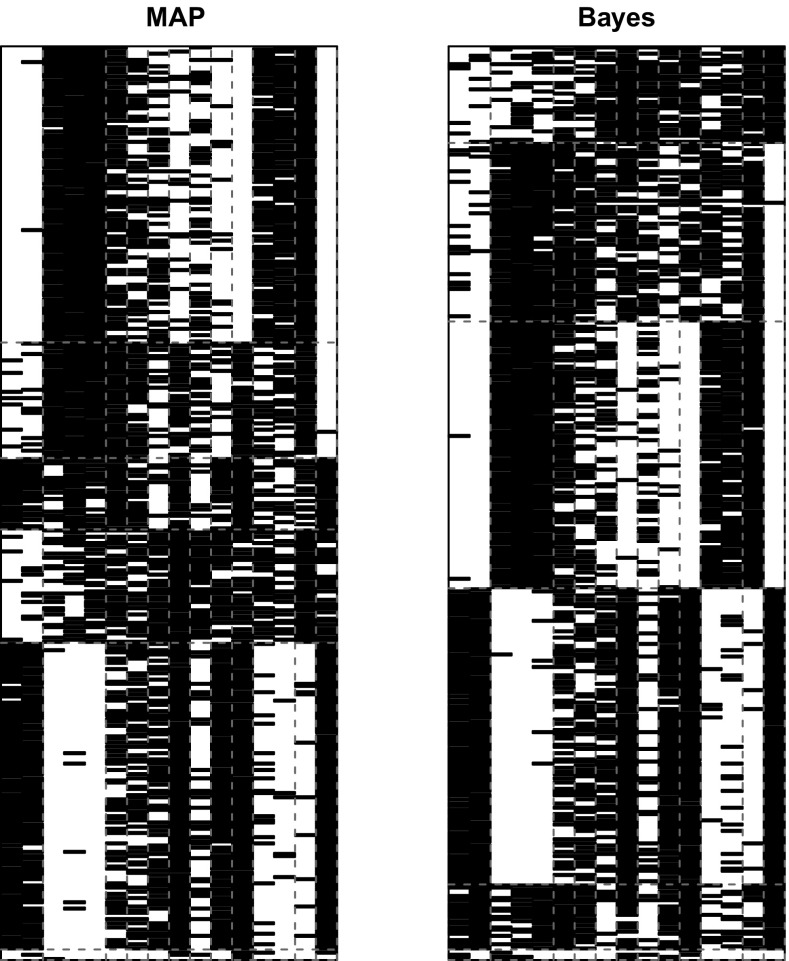
Congressional voting data. Reordered adjacency matrices for the MAP and the VI partitions. The partitions on the columns (issues) are equivalent, whereas the rows are clustered in different ways, although the number of clusters is equal

Bipartite latent block models may also be recast as mixture models, where the mixture is with respect to the partitions:

31 $$\begin{aligned} P\left( \mathcal {Y}\vert \varvec{\theta }, \Pi \right) = \sum _\mathbf{r ,\mathbf c } p\left( \mathbf r \vert \varvec{\theta } \right) p\left( \mathbf c \vert \varvec{\theta } \right) P\left( \mathcal {Y}\vert \mathbf r , \mathbf c , \Pi \right) . \end{aligned}$$

The connection probabilities $\pi _{gh}$ are realisations of independent Beta random variables for every $g=1,\dots ,K_r$ and $h=1,\dots ,K_c$, and all of the hyperparameters are fixed to 0.5.

Since conjugate priors are used, all of the model parameters can be integrated out analytically, thereby obtaining the marginal posterior $p\left( \mathbf r ,\mathbf c \vert \mathcal {Y} \right)$ in exact form. Further details on the integration can be found in Wyse and Friel (2012), Wyse et al. (2017).

#### Results

The algorithm of Wyse and Friel (2012) was used to obtain a sample for the allocations of both congress members and issues. Similarly to previous analyses, 1 million observations were discarded and 10, 000 were used as final sample using a thinning of 100. The partitioning of the data corresponding to the highest posterior value was saved as a reference. We found that posing a clustering problem on the issues was not particularly interesting in that very few issues were aggregated in the same cluster; hence, we show here only the cluster analysis on the congress members. The sample of partitions for the members was processed through the procedure of Sect. 5, and then, several runs of the greedy optimisation were performed. The computational time needed to get the sample was about 30 hours, whereas an average of 70 s was required for each run of the greedy algorithm, with $K_{\mathrm{up}}$ fixed to 30. We note that the optimal value was actually reached in $65\%$ of the runs. Figure 9 shows the posterior sample for the number of groups. The reordered adjacency matrices for the MAP and the VI-optimal partition are shown in Fig. 10. From the confusion table shown in Table 5 it appears that the two main political factions are split into three subgroups each, with a total of 29 individuals against the tide.

**Table 5:** Congressional voting data: confusion matrix comparing the political affiliation with the VI partition

	1	2	3	4	5	6
Democrat	42	79	127	16	1	2
Republican	4	6	0	125	30	3

## Conclusions

We have proposed a Bayesian approach to summarise a sample of partitions from an arbitrary clustering context. Our method relies on a greedy algorithm which is particularly efficient in finding both the optimal partition and the best number of groups by minimising the average loss with respect to a posterior sample. The method can be used in a variety of clustering contexts and it can handle many well-known loss functions.

In this paper we have specifically addressed the computational issues related to this problem, and we have shown that our algorithm can scale well with the number of items to be classified. In addition, while previous methods focused only on ad hoc criteria or particular choices of the loss function, our methodology is the only scalable method that can encompass most comparison measures within a unified framework. Furthermore, by construction, label-switching issues do not affect our method.

The greedy algorithm usually converges with very few iterations; however, several restarts may be useful to avoid convergence to local optima. In some of the applications proposed, in order to assess the rates of convergence, 100 runs of the algorithm were performed for each dataset. Unless the computational efficiency is not a priority, we advice against this practice. Convergence was excellent on one of the datasets, but it was less successful on a more challenging one. We noticed that, when compared to other similar greedy routines (Côme and Latouche 2015; Wyse et al. 2017; Bertoletti et al. 2015), the algorithm is more likely to converge to the global optimum, even though no final hierarchical merge step is used. This may be a consequence of the fact that the objective function is generally smoother and easier to optimise. We have proposed a simulation study to analyse the performances of the various loss functions and the computational costs required. The variation of information loss has been shown to perform very well in terms of model-choice, whereas the other losses considered, including Binder’s loss, tend to largely overestimate the number of groups.

The wide applicability of our algorithm comes at a cost: each step of the optimisation process involves a computational cost depending on the size of the sample *T*, which can easily make the problem intractable if a large sample is used. However, in most cases this impasse can be downsized simply by “thinning” the sample. In addition, we have proposed a generalisation of our algorithm to include weighted samples: situationally, this may allow one to decrease the number of partitions further. As concerns storage costs, the main bottleneck is set by the *T* contingency tables of size $K^2$ that are used throughout the optimisation.

To emphasise the context independence of our approach, we have proposed applications to real datasets for three different clustering frameworks. In the Gaussian mixture model case (Old Faithful dataset), the loss function used favours 4 groups, diverging from the findings of previous works. The results on the French political blogosphere also appear to be very different from those obtained through previous analyses. On the one hand an overestimation of the number of groups may be argued; on the other hand the groups obtained with our approach are evidently more homogeneous. A clustering problem on the members of the congressional voting data has also been proposed: here the two main political factions are well recognised, and the results seem to agree with the previous analyses of Wyse and Friel (2012), Wyse et al. (2017).

One limitation of our approach is that it only provides a point estimate of the best partition. While the optimal clustering summarises all of the information contained in the posterior sample, there is no direct assessment of the uncertainty associated with the optimal solution. Due to the discrete nature of the space, and due to the obvious computational difficulties, this issue is rather difficult to address and it seems not to have a straightforward solution. However, it should be noted that interesting advances on this problem have been recently made by Wade and Ghahramani (2015).

A second important drawback of our method is that it primarily relies on the quality of the posterior sample. Often, the posterior distribution of a clustering model can be extremely complex or multimodal, making the convergence of Gibbs samplers rather slow. This is discussed for example in Hastie et al. (2015). The collapsed Gibbs samplers of Nobile and Fearnside (2007), McDaid et al. (2013), Wyse and Friel (2012) are particularly computationally efficient, yet too often they suffer of high rejection rates and poor mixing. Unfortunately, at the moment there are no conclusive solutions to address this impasse, suggesting that future research should focus on introducing new ways to explore the space of partitions $\mathcal {Z}$ in a clever way, hence making MCMC approaches more efficient.
